# On the Abilities of Unconscious Freudian Motivational Drives to Evoke Conscious Emotions

**DOI:** 10.3389/fpsyg.2019.00470

**Published:** 2019-03-07

**Authors:** Michael Kirsch

**Affiliations:** Institute of Physiological Chemistry, Essen University Hospital, Essen, Germany

**Keywords:** SEEKING, affective neuroscience, orexin, Bowlbyian drive, unconscious

## Abstract

Human beings use conscious emotions to direct their behaviors. There is some agreement in the scientific community that unconscious motivations are able to evoke conscious emotions. This manuscript focuses on Freudian motivational drives as inductors for unconscious motivation, and also on Panksepp’s framework of affective neuroscience for describing the generation of emotions. Recently, it has been suggested that imperative motor factors of Freudian drives (i.e., the hormones ghrelin, testosterone, angiotensin II and adenosine) have the ability to activate both a drive-specific brain area and brain areas of the SEEKING command system. In fact, this manuscript contends that all imperative motor factors have typical SEEKING targets (i.e., so-called receptors) in the brain areas of both *nucleus accumbens* and *lateral hypothalamus*. In addition, all imperative motor factors are able to target the *central amygdala* directly, a brain area classified by Panksepp as the instinctual part of the FEAR command system. Another point of interest may be the evaluation that imperative motor factors of the sexual drive, hunger and thirst can directly activate the RAGE command system by targeting the *medial amygdala*. Surprisingly, all imperative motor factors are able to modulate Panksepp’s granddaddy mechanism, i.e., to stimulate all seven command systems via the *lateral hypothalamus*. Orexinergic neurons exclusively located in the *lateral hypothalamus* have targets for imperative motor factors and project axons to characteristic brain areas of all seven command systems. From the fact that the imperative motor factors of the sexual drive and hunger act in an excitatory manner on orexinergic neurons whereas those of thirst and sleep inhibit such neurons, temporary termination of hunger by thirst may be understood as a very simple example of a co-regulation of Freudian drives. The author wishes to note that there are motivational drives other than the ones described by Freud. Bowlby was obviously the first in describing such drives, and Bowlbyian drive activities cannot be explained with the intermediacy of imperative motor factors. Nevertheless, the ignorance of the magnificent importance of imperative motor factors must be discarded.

## Introduction

At present, any consensus on a single unitary definition of the construct of motivation, derived from the Latin words *movere* and *motivus* (Gilbert, 2015; Strombach et al., 2016), is lacking in the psychological community (Gneezy et al., 2011). Panksepp noted that a motivation can be descripted as a process, “*in which a bodily need is subserved by a behavior*” in contrast to emotions “*where no bodily need is evident*” (Panksepp, 1998, p. 228). Thus, motivation can therefore be understood as an active movement of an individual initiated by a stimulus as a driving force. Such a view intrinsically predicts that the activity of a (motivational) drive will evoke a motivation. In the past, various classic psychological drive concepts – Hull’s drive reduction theory (Hull, 1943), Lorenz’s hydraulic conception of drive (Lorenz and Leyhausen, 1973), Tinbergen’s hierarchical organization of circuit nodes (Tinbergen, 1950) and Freud’s theory of motivational drives (Freud, 1905, 1915a,b,c) – were developed in order to explain drive-dependent motivations. It should be noted that (at present) a drive cannot be experimentally distinguished from a corresponding motivation when a (Freudian) drive acts as the driving force for this motivation. Adolphs and Anderson noted “*The difference between* ‘*drive*’ *and* ‘*motivation*’ *is more of an operational and conceptual one than a biological one*.” (Adolphs and Anderson, 2018, p. 148). In the present manuscript the construct of motivation will be nevertheless advocated but the author will distinguish metabolic-deficit-dependent motivations and metabolic-deficit-independent ones. Remarkably, Freud [a man of vast reading (Solomon, 1974)] obviously picked-up the idea of “chemical messengers”, with the first hormone identified in 1902 (Bayliss and Starling, 1902). Freud respected the intermediacy of hormones in his motivational drive theory with its known four elements somatic source, aim, object and imperative motor factor (i.e., hormones) (Freud, 1905, 1915a). Very unfortunately, persistent mistranslation of the German nouns *Drang* (correctly, in the Freudian sense: imperative motor factor) as “motor factor,” *Trieb* (correctly: drive) as “instinct” and *Trieblehre* (correctly in the Freudian sense: theory of motivational drives) as “theory of instincts” have given rise to a variety of misunderstandings, especially in those cases where Freud used the German word ‘Instinkt’ which was also (correctly) translated as instinct. For Freud instincts (‘Instinkte’) are “*inherited mental formations*” (Freud, 1915c, p. 3017) whereas drives “*represent an instigation to mental activity*” (Freud, 1926b, p. 4343; Holder, 1970, pp. 19). In 1923, Freud clarified that two types of Freudian motivational drives are constituent elements of *Eros: “According to this view we have distinguish two classes of instincts, one of which, the sexual instinct or Eros, is by far the more conspicuous and accessible to study. It comprises not merely the uninhibited sexual instinct proper and the instinctual impulses of an aim-inhibited or sublimated nature derived from it, but also the self-preservative instinct,…”* (Freud, 1923, p. 3974). Thus, according to Freud, three motivational drives (sexual drive, thirst and hunger) are constituent elements of *Eros*. In order to answer Freud’s question of “*What instincts should we suppose there are, and how many?*” (Freud, 1915a, p. 2961), we advocated three criteria for identifying a Freudian motivational drive: an imperative nature of the drive as a psychological criterion, orchestration via the *lateral hypothalamus* as a neurobiological cachet and a drive termination by means of the central release of 5-hydroxytryptamine as a biochemical attribute (Kirsch and Mertens, 2018). By using these criteria, we identified the sexual drive, thirst, hunger (in line with Freud’s prediction) and sleep as Freudian motivational drives with the corresponding imperative motor factors testosterone, angiotensin II, ghrelin and adenosine. These hormones address the frequently ignored problem of drive specificity – “*The ability to process and* ‘*decide*’ *between the drives might be lost if each drive is not also an independent generator. In other words, we have to sustain drive-specificity…*” (Wright and Panksepp, 2012, p. 18) – because they can simultaneously activate a drive-specific brain area and typical brain areas that are responsible for seeking of resources (*vide infra*).

In contrast to motivations, a variety of very detailed theories for describing emotions have been outlined so far, i.e., the Appraisal Theory (Scherer, 1984, 2009; Lazarus, 1991), Interoceptive Theories (Damasio, 1999; Craig, 2002; Damasio and Carvalho, 2013), Constructed Emotion Theory (Feldman Barrett, 2017), Theory of Emotion (Rolls, 1999), Higher-Order Theory of Emotion (LeDoux and Brown, 2017) and Emotion Systems (Panksepp, 1998; Panksepp and Biven, 2012). The latter framework should be attractive from the perspective of Freudian motivational drives because of the fact that Panksepp’s theory of affective neuroscience tends to emphasize motor-related representations (i.e., drives) in the development of feelings. Panksepp (Panksepp, 1998) classifies seven different types of command systems that may (but do not necessarily have to) evoke special behaviors, e.g., *seeking* for rewards/resources/sexual partners, *lust*, *caring* and affection, loss and *panic*, *rage*, *fear* and *play*. Special subcortical regions of the brain are involved with the processing to the corresponding conscious emotions,^1^^,^^2^ which are classified as so-called command systems (labeled SEEKING, RAGE, FEAR, LUST, CARE, PANIC, and PLAY) (Panksepp, 1998, 2016, 2018; Watt and Panksepp, 2009; Zellner et al., 2011; Solms and Panksepp, 2012; Wright and Panksepp, 2012; Panksepp and Yovell, 2014; Alcaro et al., 2017). Since an activated SEEKING system constantly blends well with all the other command systems by co-regulating them (Wright and Panksepp, 2012), the generation of SEEKING activities (according to Panksepp the SEEKING system is “*the ‘granddaddy’ of all the emotional system.*” (Panksepp and Biven, 2012, p. 86), are of central importance in the development of conscious emotions. Recently, we were able to contend the unexpected possibility that the SEEKING system can be activated by the intermediary action of Freudian imperative motor factors (Kirsch and Mertens, 2018). Since Freud explicitly noted that a motivational drive has an unconscious nature: “*I am in fact of the opinion that the antithesis of conscious and unconscious is not applicable to instincts. An instinct can never become an object of consciousness* – *only the idea that represents the instinct can.*” (Freud, 1915c, p. 3000) the question arose how an unconscious stimulus can evoke a conscious sensation? From findings of our last work it is possible to expand a fine idea introduced by Panksepp *et* Biven (Table 1).

**Table 1:** Types of chemical messenger codes.

Type of	Unconscious	Conscious^a^	
Processing	Freudian Drive	AFFECTIVE^a^	COGNITIVE^a^
Signaling code	Hormone codes	Neuromodulator codes^a^	Neurotransmitter codes^a^


*^*a*^According to (Panksepp and Biven, 2012, p. 8).*

Thus, according to Panksepp, the intermediacy of neuromodulators and/or classical neurotransmitters represents a mandatory premise for consciousness.^3^ Since it has been mentioned that there are qualitative differences of the nervous system between conscious and unconscious processes (Brakel and Shervin, 2005) and because Freudian drives have an unconscious nature (vide supra) and uses hormones as signal transporters, the unconscious action of the Freudian drives can be determined biochemically at the level of signaling codes (Table 1).^4^ For example, an imperative motor factor generated outside the brain [e.g., stomach derived release of ghrelin (Kojima et al., 1999; Stievenard et al., 2017)] cannot be transformed to a neurotransmitter/neuromodulator (i.e., cannot be transformed to signals necessary to achieve consciousness) as long as it circulates in the periphery. After passing the blood-brain barrier, the imperative motor factor can now induce the release of neurotransmitters/neuromodulators by occupying its hormone receptors on various pre-synapses in subcortical brain areas. This release of neuromodulators or neurotransmitters represents – from the perspective of the Global Neuronal Workspace Hypothesis (Dehaene et al., 2006) – the provision of preconscious stimuli that can (but do not necessarily have to) gain access to conscious processing.

Unfortunately, the abilities of imperative motor factors to address the subcortical brain in such a manner, was only deconvolved for the brain areas of the SEEKING system (Kirsch and Mertens, 2018). This option raises the question of whether imperative motor factors have the ability to modulate activities of other command systems, and – if so – the underlying mechanisms would be of interest. This manuscript will report on such totally underestimated capabilities, although they are quite well evaluated.

## Direct Mechanisms for the Generation of Command System Activities

The idea of making a direct connection between Freudian drives via their imperative motor factors and Panksepp’s emotional command systems intrinsically required one to locate targets (i.e., so-called receptors) of these hormones at the brain areas of interest (Table 2).

**Table 2:** Targets of imperative motor factors on brain areas of command systems.

Affective prototype and brain areas^a^	Ghrelin Hunger	Testosterone Sexual drive	Angiotensin II Thirst	Adenosine Sleep
SEEKING				
LH	Reference 1	Reference 2	Reference 3	Reference 4
NAc	Reference 5	Reference 6	Reference 7	Reference 8
VTA	Reference 9	Reference 10	–	Reference 11
RAGE				
MeA	Reference 12	Reference 13	Reference 14	–
FEAR				
CeA	Reference 15	Reference 16	Reference 17	Reference 18
BLA	Reference 19	–	–	Reference 20
LUST				
VMH	Reference 21	Reference 22	–	–
CoA	–	Reference 23	–	–
CARE				
BNST	–	Reference 24	–	–
PANIC				
BNST	–	Reference 24	–	–
AnT	–	–	–	References 11 and 17
PLAY				
CmT, DmT, PT	–	–	Reference 25	–


****^*a*^**classification according to Watt (Watt, 2017), AnT, *anterior thalamus*; BLA, *basolateral amygdala*; BNST, *bed nucleus of stria terminalis*; CeA, *central amygdala*; CmT, *centromedian thalamus*; CoA, *cortical amygdala*; DmT, *dorsomedial thalamus*; LH, *lateral hypothalamus*; MeA, *medial amygdala*; NAc, *nucleus accumbens*; PT, *posterior thalamus*; VMH, *ventromedial hypothalamus*; VTA, *ventral tegmental area*. Reference 1, (Mitchell et al., 2001; Toshinai et al., 2003); Reference 2, down-stream product of testosterone (Shughrue et al., 1997; Muschkamp et al., 2007); Reference 3, (Yoshida et al., 2012); Reference 4, (Svennigsson et al., 1997; Thakhar et al., 2002); Reference 5, (Egecioglu et al., 2010); Reference 6, (Cunningham et al., 2012); Reference 7, (Jenkins et al., 1997; Mendelsohn et al., 1984); Reference 8, (Svennigsson et al., 1997; Rosin et al., 2003; Ferre et al., 2007); Reference 9, (Zigman et al., 2006); Reference 10, (Simerly et al., 1990); Reference 11, (Svennigsson et al., 1997); Reference 12, (Alvarez-Crespo et al., 2012); Reference 13, (Simerly et al., 1990; Cunningham et al., 2012; He et al., 2013); Reference 14, (Lenkei et al., 1996); Reference 15, (Cruz et al., 2013; Yoshimoto et al., 2017); Reference 16, (Simerly et al., 1990); Reference 17, (Lenkei et al., 1996); Reference 18, (Goodman and Snyder, 1982); Reference 19, (Alvarez-Crespo et al., 2012; Yoshimoto et al., 2017); Reference 20, (Svennigsson et al., 1997; Rau et al., 2014, 2015; Simoes et al., 2016); Reference 21, (Zigman et al., 2006); Reference 22, (Simerly et al., 1990; Cunningham et al., 2012; He et al., 2013); Reference 23, (Simerly et al., 1990); Reference 24, (Cunningham et al., 2012; He et al., 2013); Reference 25, (Mendelsohn et al., 1984; Lenkei et al., 1996).**

As expected from evaluations of our earlier manuscript (Kirsch and Mertens, 2018), all imperative motor factors can directly generate SEEKING activities because, in addition to targets in their drive specific brain areas^5^, they all also have anchorage grounds in both the *lateral hypothalamus* and the *nucleus accumbens* (Table 2). The activation of neurons in the latter area and also in the *ventral tegmental area* can result in the release of the catecholamine dopamine (Naleid et al., 2005; Abizaid et al., 2006; Jerlhag et al., 2007), and that neuromodulator is a key intermediate in the activation of the SEEKING system (Panksepp, 1998; Panksepp and Biven, 2012; Watt, 2017).

Somewhat surprising was the evaluation that all imperative motor factors have receptors in the *central amygdala*, a brain area that was classified as a part of the FEAR command system (Panksepp, 1998; Panksepp and Biven, 2012; Watt, 2017). Thus, all imperative motor factors have direct access to both the SEEKING and the FEAR command systems. Panksepp noted that a variety of chemical messengers can activate the FEAR system (Panksepp and Biven, 2012), with the result that the view that imperative motor factors can provide a similar activation is not in conflict with the theory of affective neuroscience. In addition, Panksepp distinguished between conditional FEAR and unconditional ones, stating “*Therefore, while the central nucleus of the amygdala is part of the unconditional (instinctual) FEAR system, the other nuclei are not.*” (Panksepp and Biven, 2012, p. 196). Therefore, the action of the imperative motor factors on the INSTINCTUAL FEAR system may represent a phylogenetic old mechanism.^6^ Of course, as most Freud followers would expect, Freud was aware of a link between his motivational drives and fearful emotions: “*So far we have had no occasion to regard realistic anxiety in any different light from neurotic anxiety. We know what the distinction is. A real danger is a danger which threatens a person from an external object, and a neurotic danger is one which threatens him from an instinctual demand*.” (Freud, 1926a, p. 4319). The fact that imperative motor factors have direct access to the FEAR command system by targeting the *central amygdala* may also be an interesting finding for psychoanalysts of other schools because Bowlby noted. “*No CONCEPT is more central to psychoanalytical theory than the concept of anxiety. Yet it is one about which there is little consensus of opinion, which accounts in no small measure for the divisions between different schools of thought. Put briefly, all analysts are agreed that anxiety cannot be explained simply by reference to external threat: in some way processes usually thought of as internal and instinctive seem to play a crucial role. But how these inner forces are to be conceptualized and how they give rise to anxiety has always been a puzzle.*” (Bowlby, 1960). In summary, direct access of Freudian drives to the FEAR command system is not in conflict with either the theory of affective neuroscience or historical predictions by leading psychoanalysts.

Of the other command systems, only RAGE can be addressed via the *medial amygdala* by three imperative motor factors. The failure of adenosine, i.e., the imperative motor factor of sleep, to activate RAGE can be expected because ongoing *rage* is obviously counterproductive for the onset of sleep, and therefore such neurochemicals that can be elevated during RAGE (e.g., noradrenaline) are decreased during sleep and *vice versa* (Watson et al., 2010; Panksepp and Biven, 2012).

Although there are (beyond any doubt) direct mechanisms for the generation of command systems activities, their capability is somewhat limited, as imperative motor factors cannot evoke all types of emotion via that mechanism with the same level of efficiency. Therefore, the question arose as to whether Freud’s imperative motor factors can even do more than generate SEEKING, FEAR and RAGE activities by occupying a receptor in a typical brain region of these command systems.

## Indirect Mechanisms for the Generation of Command System Activities

Since all Freudian drives are orchestrated via the *lateral hypothalamus* (*vide supra*), the precise targets of imperative motor factors in this brain area have been analyzed (Table 3).

**Table 3:** Targets for imperative motor factors of hunger, sexual drive, sleep and thirst on orexinergic neurons located in the *lateral hypothalamus.*

Receptor/imperative motor factor	Action on OX neurons	Reference

GHS-R/Ghrelin	Excitatory	Mitchell et al., 2001; Toshinai et al., 2003
AR/Testosterone	Excluded	Simerly et al., 1990; Muschkamp et al., 2007
ER/Estrogen	Excitatory	Shughrue et al., 1997; Muschkamp et al., 2007
A_1_R/Adenosine	Inhibitory	Svennigsson et al., 1997; Thakhar et al., 2002
AT_1a_R/Angiotensin II	Inhibitory	Yoshida et al., 2012


*OX, orexinergic; GHS-R, growth hormone secretagogue receptor; AR, androgen receptor; ER, estrogen receptor; A_*1*_R, adenosine receptor type 1; AT_*1A*_R, angiotensin II receptor type 1A.*

This analysis contains two surprises. Firstly, attractive targets for testosterone on orexinergic neurons are lacking in the *lateral hypothalamus* (Table 3), although androgen receptors have been detected in that area (Simerly et al., 1990). Beside testosterone, its downstream product estradiol – the enzyme aromatase directly oxidizes testosterone into the estrogen derivative estradiol (Fui et al., 2014) – is also important for male sexual behavior (Cunningham et al., 2012), and estrogen receptors on orexinergic neurons are present in the *lateral hypothalamus*. The second surprise was the realization that receptors of all four imperative motor factors modulate (in an inhibitory or excitatory manner) the release of the neuromodulator orexin (Table 3). In 1998, two research groups independently identified peptides exclusively produced by neurons located in the *lateral hypothalamus* (de Lecea et al., 1998; Sakurai et al., 1998). Most scientific journals have now accepted the name “orexins” (instead of the alternative designation “hypocretins”) for these peptides, although their physiological actions are not limited to the regulation of appetite (Li et al., 2014). Very interestingly, from their location in the *lateral hypothalamus*, orexinergic neurons project axons to brain areas that are important for the seven command systems of affective neuroscience. In detail, orexinergic neurons project to brain areas related to SEEKING [*ventral tegmental area* (Marcus et al., 2001; Uramura et al., 2001; Korotkova et al., 2003; Borgland et al., 2008; Muschkamp et al., 2014) and *nucleus accumbens* (Trivedi et al., 1998; Marcus et al., 2001; Smith et al., 2002; Yang and Ferguson, 2003)], RAGE [*medial amygdala* (Trivedi et al., 1998; Marcus et al., 2001) and *bed nucleus of stria terminalis* (Trivedi et al., 1998; Conrad et al., 2012; Lungwitz et al., 2012)], FEAR [*central amygdala* (Marcus et al., 2001; Cluderay et al., 2002; Bisetti et al., 2006) and *basolateral amygdala* (Arendt et al., 2014)], LUST [*ventromedial hypothalamus* (Muroya et al., 2004)], CARE [*ventral periaqueductal gray matter* (Li et al., 2014)], PANIC [*midline thalamic nuclei* (Bayer et al., 2002; Ishibashi et al., 2005), *paraventricular thalamus* (a midline thalamic structure) (Peyron et al., 1998; Marcus et al., 2001; Huang et al., 2006; Li et al., 2011) and *mediodorsal thalamus* (Govindaiah and Cox, 2006)] and finally PLAY [*parafascicular nucleus* (Govindaiah and Cox, 2006)]. The postsynaptic action of orexin is generally an excitatory one on other neurons and therefore leads to the release of a variety of either neurotransmitters or neuromodulators. For example, the release of orexin in the *ventral tegmental area* stimulates dopaminergic (Marcus et al., 2001; Uramura et al., 2001; Korotkova et al., 2003; Muschkamp et al., 2014) as well as non-dopaminergic neurons (Korotkova et al., 2003; Borgland et al., 2008). Some of the aforementioned brain areas have projections to the *lateral hypothalamus*, thereby offering an afferent regulation of orexinergic neurons (as a kind of a feedback mechanism^7^). For instance, neurons of the *medial amygdala* (RAGE) project to orexinergic neurons of the *lateral hypothalamus* (James et al., 2017). However, knowledge of these afferent projections is currently too low to permit detailed insight into such feedback mechanisms.

The fact that orexinergic neurons of the *lateral hypothalamus* project to brain areas of all command systems supports Panksepp’s postulation that the SEEKING system, the *lateral hypothalamus* having been classified as a part of this command system (Watt, 2017), is “*the ‘granddaddy’ of all the emotional system.*” (Panksepp and Biven, 2012, p. 86). Since the author is unaware whether other neurons can act in a similar manner (but, of course, cannot exclude such a possibility with certainty), and because the activity of orexinergic neurons is under the control of Freud’s imperative motor factors (Table 2), it is concluded that the activity of the granddaddy mechanism, i.e., to evoke emotions via generation of SEEKING-dependent command system activities, can be under the control of Freudian motivational drives.^8^

It is well known that different motivations can co-regulate (conflict or support) each other (Huang and Bargh, 2014; Gilbert, 2015). Such a co-regulation of Freudian drives can be understood at a biomolecular level with the aid of Table 3 because hunger and the sexual drive can stimulate command systems activities by enhancing orexin-dependent networking, whereas thirst and sleep can operate oppositely. The sense of this mechanism should be illustrated by answering the question what drive must a hungry and thirsty person firstly satisfied? Mahatma Gandhi survived (three times) 21 days of complete starvation (Chettiar, 1943; Gandhi, 1948) but an average human being can probably survive without water for about only a few days (Kottusch et al., 2009). Thus, from a perspective of survival, the satisfaction of thirst is the more important one, and the thirst drive is able to counteract the claim of the hunger drive by downregulating orexin-dependent activation of the seven command systems. In fact, there is data to support the assertion that the claim of thirst is stronger and more stable over the day compared to the claim of hunger (Mattes, 2010). Temporary termination of the claim of the hunger drive by thirst is reminiscent of the phenomenon of a Freudian repression “*the impulse then passes into the state of ‘repression’ [Verdrängung].*” (Freud, 1915b, p. 2977), but albeit the expressed example is far too simple for describing such a complex psychological entity.^9^ Thus, the importance of the mechanism of drive co-regulation via orexin-mediated networking for psychological entities cannot be classified at present.^10^

## Limits of Imperative Motor Factors for the Generation of Affective Neuroscience Activities

Although the indirect pathway – i.e., modulation of orexin-dependent networking by targeting the *lateral hypothalamus* (Table 3) – expands the capabilities of imperative motor factors, they are unable to cover all possible types of motivations and (corresponding) emotions. A Freudian motivational drive is down-regulated by the cerebral release of 5-hydroxytryptamine (*vide supra*). Therefore, drives and corresponding motivations that would require the intermediacy of 5-hydroxytryptamine for their processing cannot be regarded as being dependent upon Freudian motivational drives. This gives rise to the puzzler: Do such drives really exist, and where are they operating in psychological situations? Most surprisingly, an entirely unexpected answer is offered by Bowlby’s attachment theory (Bowlby, 1960, 1973). The unconscious motivation of an infant to stay in close proximity to its care provider becomes measureable by expanding the distance between mother and infant for significant periods of time, resulting in distress, anxiety and fear in the infant (Bowlby, 1973). Bowlby mentioned that a drive (even suggested by Freud) is responsible for this motivation of the infant: “*Our most conservative conclusion is that Freud was not wholly satisfied with his earlier accounts* [i.e., theory of motivational drives]. *A more radical one is that, toward the end of his life and imbued with a newly-found but vivid appreciation of the central importance of the child’s tie to his mother, Freud was not only moving away from the theory of Secondary Drive* [i.e., motivational Freudian drive] *but developing the notion that special drives built into the infant in the course of evolution underlie this first and unique love relationship.”* (Bowlby, 1958). Of course, Bowlby’s suggestion of classifying a Freudian drive as a Secondary Drive evoked a number of heavy protests from leading Freud followers, but the one proffered by Anna Freud points to a hitherto unrecognized solution: “*He* [Bowlby] *sets up a controversy between the tie to the mother and the action of the pleasure principle in terms of “primary and secondary drive” and criticizes us for reversing their order of importance*, i.e., *for regarding the tie to the mother as a secondary, the search for pleasure as a primary instinctual urge.*” (Freud, 1960). Most remarkably, Anna Freud accepted Bowlby’s view that there are other drives at work as well as the motivational Freudian ones. It should therefore be helpful to classify these non-Freudian drives as Bowlbyian ones^11^. The dispute concerning the ranking of the drives is presumably futile since both kinds of drives are obviously essential for the survival of the human species. Thus, by accepting the view that the motivation of the child to stay in close proximity to its mother is the result of a Bowlbyian drive activity, a detailed search in literature would be of interest with regard to the possibility of whether 5-hydroxytryptamine is involved in motivations/emotion connected with attachment in general. The observation that a polymorphism of the 5-HT_2A_ serotonin receptor gene – this receptor being one important target for 5-hydroxytryptamine in the brain – is connected with the psychological disorder referred to as ‘avoidant attachment’ (Gillath et al., 2008) currently offers the strongest proof that 5-hydroxytryptamine is in fact involved in the processing (and not in the down-regulation^12^) of a Bowlbyian drive. In addition, 5-hydroxytryptamine increases the secretion of oxytocin (Saydoff et al., 1991; Bagdy and Makara, 1994) and this neuromodulator is obviously highly important for the tie between an infant and its mother (Uvnäs Moberg and Prime, 2013). Since 5-hydroxytryptamine down-regulates the activity of a Freudian drive initiated by imperative motor factors and because 5-hydroxytryptamine supports via increase of oxytocin secretion the processing of a Bowlbyian drive, it can be safely concluded that imperative motor factors are not responsible for Bowlbyian drive activities. Of course, the lack of knowledge of how an impaired or down-regulated Bowlbyian drive is able to activate command system activities – according to Panksepp, an impaired attachment activates the PANIC/GRIEF system (Panksepp and Biven, 2012, pp. 312–313) – needs to be evaluated.

## Failure of Emotions to Initiate Freudian Drive Dependent Motivations

Experimental psychologists demonstrate that both positive emotionally valent stimuli and negative (aversive) ones can successfully enhance (the motivation) ‘attention’ in patients (Dominguez-Borras et al., 2013; Vuilleumier, 2015). From such experimental findings psychologists have concluded that emotions provide guidance for motivations and are linked with them (e.g., Gilbert, 2014). However, the conclusion that an emotion can initiate a motivation has hardly any means of little significance, given the lack of a generally accepted definition of the term ‘motivation’ in psychology. Since an emotion can inform an individual about the existence of a metabolic deficit (but cannot be responsible for such an imbalance), it is concluded that an emotion cannot initiate a Freudian drive dependent motivation. The expressed example, the putative motivation ‘attention’ is obviously independent of the intermediary action of a Freudian drive and would be (as long as any generally accepted definition of the term ‘motivation’ is lacking) tentatively classified as a metabolic deficit independent motivation that might be liberated by an emotion. The next puzzler, namely whether an emotion can provoke a Bowlbyian drive dependent motivation, cannot be answered yet because the precise architecture of this complex drive still needs to be evaluated.

## Conclusion

**FIGURE 1:**
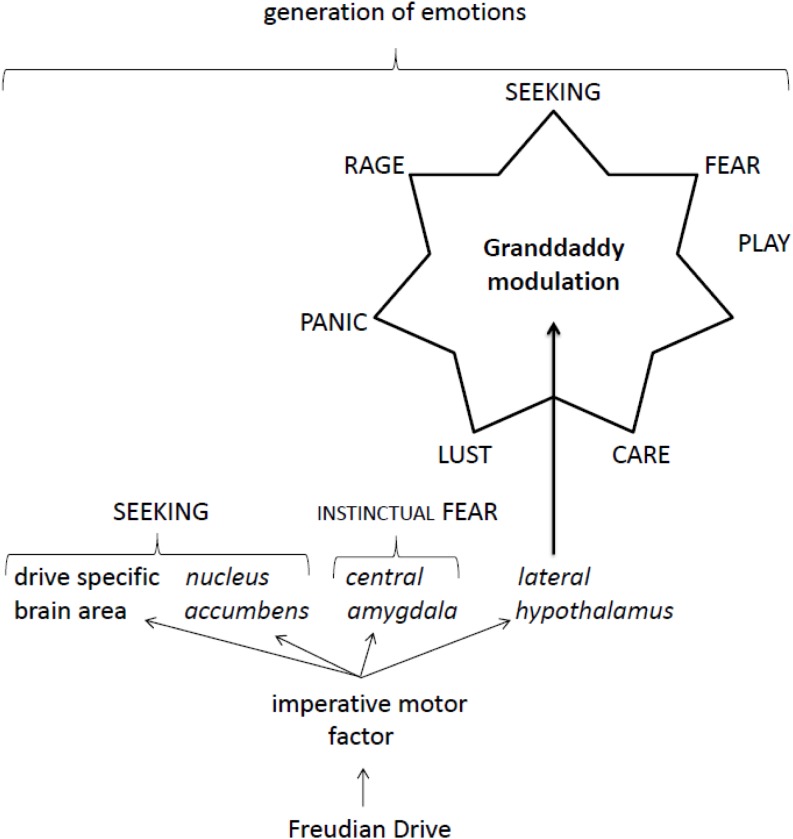
Proposed action of Freudian drive-dependent generation of emotions.

This manuscript has been written under the assumption that our recently published update (Kirsch and Mertens, 2018) to Freud’s 100-year-old (but essentially accurate) theory of motivational drives needs to be conclusively expanded in order to exploit its full potential. Not just the SEEKING system, but imperative motor factors of all Freudian drives have targets in the *central amygdala* (Table 2), a brain area that was classified by Panksepp (Panksepp and Biven, 2012, p. 196) as the instinctual part of the FEAR command system. In addition, the imperative motor factors of the sexual drive, hunger and thirst also have targets in the *medial amygdala* (Table 2), an area of the brain classified as being part of the RAGE command system (Watt, 2017). Thus, besides directly generating SEEKING activities, all imperative motor factors are able to generate FEAR activities, and three of them can also directly stimulate the generation of RAGE activities. In addition, all drives can indirectly modulate all sorts of command system activities by controlling Panksepp’s ‘granddaddy’ of affective neuroscience, i.e., by modulating the activity of orexinergic neurons in the *lateral hypothalamus* (Table 3). Because of this, the sexual drive and hunger can stimulate affective neurophysiological activities via orexin-mediated networking, whereas sleep and thirst can inhibit such demands. The opposing actions of hunger and thirst were first used to explain the co-regulation of a Freudian drive. In order not to overrate the deconvolved impressive capabilities of Freudian drives, the astute reader needs to note that essential motivational drive activities described by Bowlby, classified here as Bowlbyian drives, cannot be explained by the intermediacy of imperative motor factors. The action of imperative motor factors is basically drafted (Figure 1).

The consideration of actual findings on Freud’s theory of motivational drives (evaluated here and in our previous manuscript) leads to the following assertions:

(1) Human beings are directed, but not determined, by Freudian drives in an unconscious manner.
(1) The satisfaction of a Freudian drive leads to the release of the neurotransmitter 5-hydroxytryptamine in order to down-regulate the drive.
(1) The sexual drive, hunger, thirst and sleep are Freudian drives with an imperative character.
(1) The imperative motor factor of a Freudian drive is a signal molecule that directly targets *nucleus accumbens*, *lateral hypothalamus*, *central amygdala* and a drive-specific brain area.
(1) The imperative motor factor of a Freudian drive can directly evoke generation of drive-specific SEEKING and INSTINCTUAL FEAR activities.
(1) The imperative motor factors of the sexual drive, hunger and thirst also directly target the *medial amygdala*, thereby evoking the generation of RAGE activities.
(1) All imperative motor factors are able to modulate, indirectly, the generation of affective neuroscience activities by targeting orexinergic neurons in the *lateral hypothalamus*.

In summary, it can be said that the intermediacy of Freudian imperative motor factors can explain convincingly the modulation of command system activities. Accordingly, the concept of Freudian motivational drives is somewhat underestimated even by Freudian psychoanalysts, although Boag recently mentioned the putative importance of the drives for the *id* and the *ego*, respectively (Boag, 2014).

## Author Contributions

The author wrote and designed the manuscript.

## Conflict of Interest Statement

The author declares that the research was conducted in the absence of any commercial or financial relationships that could be construed as a potential conflict of interest.

## Footnotes

1 The description of the development of consciousness actually represents a multifaceted problem (e.g., Solms, 1997, 2019; Dehaene et al., 2006). In the present manuscript the author follows the view that core brainstem consciousness is the primary type of consciousness, i.e., so-called affective consciousness (Panksepp, 1998; Damasio, 2018). In any case (affective consciousness and cortical ones), consciously experienced emotions emerged from preconscious processing (Panksepp, 1998, p.34). The Global Neuronal Workspace Hypothesis can distinguish between subliminal, preconscious and conscious processing (Dehaene et al., 2006). Accordingly, a preconscious stimulus that is stored in a so-called temporary preconscious buffer, might achieve conscious state once the central workspace is released to the stimulus.

2 A group of modern psychoanalysts has made a distinction between self-conscious emotions and basic conscious emotions. Only the latter type is imbedded in biological determined action tendencies and can therefore be described using Panksepp’s Emotion Systems (Tracy et al., 2007; Schalkwijk, 2018).

3 Classical neurotransmitters are released from a special store (so-called synaptic vesicles) into a synaptic cleft, an area between two neighboring synapses (called referred to as the pre-synapse and post-synapse). The neurotransmitter signaling is completed by occupying targets (so-called receptors) on the post-synapse. Neuromodulators can be viewed as neurotransmitters of special sort because they signal between neurons in a more diffuse fashion and typically modulate the action of classical neurotransmitters by tuning the receptor affinities on the post-synapse. In addition, some neuromodulators can evoke the release of classical neurotransmitters on the pre-synapse (*vide infra*). The hormones of interest (the advocated Freudian imperative motor factors) can (in contrast to neurotransmitters and neuromodulators) be synthesized and released outside the brain and communicate between two organs. After passing the blood brain barrier they can induce the release of neurotransmitters and neuromodulators, respectively, by occupying their targets (hormone receptors) on the pre-synapses. A more detailed description of neuro-chemicals can be found in textbooks, e.g., (Nieuwenhuys, 1985; Webster and Stanford, 2001).

4 Noticeably, the translation of Freud’s term “*unbewusst*” as “unconscious” (instead of “subconscious”) confuses neuroscientists, since “unconscious” refers in medicine to brain activities during senseless states. As a result, neuroscientists tend to replace the term unconscious with either “not-conscious” or “non-conscious”. Anderson and Adolphs (Adolphs and Anderson, 2018, p. 298) noted, that the term unconscious is frequently used for unawareness of an external stimulus at subliminal levels, e.g., (Shervin and Fritzler, 1968; Greenwald et al., 1996). In contrast, by using an operating Freudian drive as a typical example of unconsciousness, it can be assumed that an unconscious action requires an internal stimulus –, and not an external one –, in order to initiate a mental process in a subconscious manner (*vide supra*).

5 The drive specific brain areas are: *arcuate nucleus* (hunger); *subfornical organ, area postrema* and *organosum vasculosum of lamina terminalis* (thirst); *medial preoptic area* (sexual drive at least in rodents); *tuberomammillary nucleus* (sleep); (Kirsch and Mertens, 2018).

6 For instance, one may speculate that a thirsty rat that can activate the INSTINCTUAL FEAR system via the intermediacy of angiotensin II, i.e., the imperative motor factor of thirst, in order to “use” the corresponding emotion *fear* to rule out the potential presence of a predator at its place of drinking, has an advantage in the struggle for survival. In fact, there is general consensus that the amygdala conciliates survival-mediated behavior (LeDoux et al., 1988; Kapp et al., 1990; Amaral et al., 1992; Fanselow, 1994; Davis, 2000; Lang and Bradley, 2018).

7 In order to discard the view that SEEKING exclusively instructs the other command systems, it seems necessary to demonstrate communication possibilities from the other command systems to SEEKING.

8 By expanding Panksepp’s analogy of “granddaddy” the writer of this manuscript comes to the end that the granddaddy lies (sometimes) on Freud’s couch.

9 One reviewer noted: “*In Freudian thinking, repression due to conflict happens because the action associated with the drive is threatening to the ego, which must refer to a threat of the ‘self’-organization of the mind, rather than due to a competing demand that is more important.*”

10 Noticeably, Freud expanded the construct of ‘repression’ to affective emotions: “*In the first place*, *it may happen that an affective or emotional impulse is perceived but misconstrued. Owing to the repression of its proper representative it has been forced to become connected with another idea, and is now regarded by consciousness as the manifestation of that idea. If we restore the true connection, we call the original affective impulse an ‘unconscious’ one. Yet its affect was never unconscious; all that had happened was that the idea had undergone repression.*” (Freud, 1915c, p. 3001). Here, Freud’s standing “*regarded by consciousness as the manifestation of that idea*” may be explained by a (partly) cortical processing and in such a case the occurrence of ‘repression’ cannot be solely explained by orexin-dependent subcortical networking.

11 Such a classification would additionally prevent confusions with Panksepp’s ‘Nested Brain Mind Hierarchies’ labeled primary-process, secondary-process and tertiary-process (Panksepp and Biven, 2012; Watt, 2017).

12 Provided that 5-hydroxytryptamine acted like in the Freudian drives as a termination signal, a Bowlbyian drive with a defect 5-HT_2A_ receptor would be over-stimulated, in contrast to the observation.
